# Manganese management in plants: Golgi transporter determines manganese allocation and cell wall composition

**DOI:** 10.1093/plphys/kiac429

**Published:** 2022-09-19

**Authors:** Stefanie Wege

**Affiliations:** Institute of Crop Science and Resource Conservation (INRES), Rheinische Friedrich-Wilhelms-Universität Bonn, D-53113 Bonn, Germany

Manganese (Mn) is an essential plant micronutrient, and Mn deficiency can be a major limiting factor for crop yield, particularly in dry, well-aerated, and alkaline soils. For example, Mn deficiency is prevalent in Texas, Northern Europe, China, and large areas in southern Australia. In addition, Mn deficiency frequently occurs in combination with other micro(nutrient) deficiencies, such as iron (Fe), making it often challenging to identify among the array of other deficiency symptoms (Broadley et al., 2012; Alejandro et al., 2020). Yet, ensuring optimal Mn nutrition is pivotal for plant growth, development, and reproduction.

The best-known role of Mn in plants is in the oxygen-evolving complex of photosystem II, where it is part of the Mn_4_Ca cluster that oxidizes and subsequently splits water molecules. One of the first Mn-deficiency symptoms in many plants is therefore leaf chlorosis (Broadley et al., 2012; Alejandro et al., 2020). Apart from its role in photosynthesis, Mn ions (Mn^2+^) are essential cofactors for different enzymes, including the Mn^2+^-dependent glycosyltransferases that synthesize cell wall precursors. These enzymes are Golgi-resident proteins that produce, among others, pectin precursors, which are then delivered to the apoplast. The Golgi localization of the Mn^2+^-dependent glycosyltransferases requires Mn^2+^ import into the lumen of this endomembrane compartment. A Mn^2+^-permeable ATPase called ER localised Calcium pump (ECA3) has been previously identified as a candidate for Mn^2+^ import into the Golgi lumen, and Arabidopsis (*Arabidopsis thaliana*) *eca3* knockout plants show severe chlorosis under Mn^2+^ deficiency (Mills et al., 2008). This suggests that chloroplasts might be primarily affected in *eca3* plants, hinting at the complexity of endomembrane Mn^2+^ transport and allocation.

**Figure 1:**
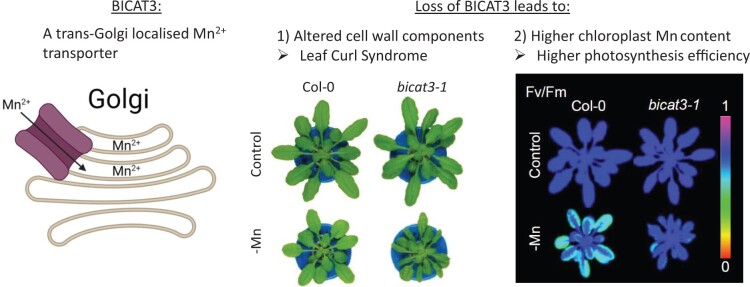
The BICAT3 can transport Mn^2+^ and is localized to trans-Golgi cisternae. Loss of BICAT3 impacts cell wall matrix polysaccharide biosynthesis in the Golgi and leads to defective cell wall component linkage and composition, resulting in Leaf Curl Syndrome. Additionally, *bicat3* plants take up more Mn^2+^, which is allocated to chloroplasts, improving photosynthesis. Partly created using biorender, adapted from He et al. (2022).

In this issue of *Plant Physiology*, (He et al. (2022), characterized the Arabidopsis Bivalent Cation Transporter 3 (BICAT3) as a principal manganese transporter localized specifically in trans-Golgi cisternae (Figure 1). They showed that BICAT3 is ubiquitously expressed in both areal and nonareal tissues, and that its function is crucial for pollen tube growth and for plant performance under Mn^2+^ deficiency, confirming and expanding on previous work (Hoecker et al., 2020; Zhang et al., 2021). Under control conditions, *bicat3* knockout plants developed similarly to wild-type plants during vegetative growth, but *bicat3* siliques were shorter. Semi in vivo pollen tube growth assays using excised pistils demonstrated that the *bicat3* male gametophyte is defective, and pollen tubes showed a strong growth defect.

While vegetative growth was similar to wild-type under control conditions, plants without BICAT3 developed severe phenotypic alterations under Mn^2+^ deficiency. Monosaccharaide analysis and sugar linkage determination revealed that *bicat3* plants had substantial modifications of cell wall matrix components compared to the wild-type. These cell wall alterations had developmental consequences, and *bicat3* plants displayed strong anatomical deformities under low Mn^2+^ conditions, such as Leaf Curl Syndrome.

Intuitively, one might assume that the phenotype of *bicat3* plants is connected to lower Mn^2+^ content in plants. Yet, changes in endomembrane ion transport often lead to surprising and unexpected downstream effects, and connecting transporter function to phenotype is often challenging (David et al., 2019). The phenotype of *bicat3* plants is no exception.

He et al. (2022) found that the loss of BICAT3 led to higher Mn^2+^ concentration in shoots under Mn deficiency, and photosynthesis was improved in *bicat3*. Mn^2+^ analysis of isolated chloroplasts revealed higher chloroplast Mn^2+^ content, strongly suggesting that this is the reason for the higher overall shoot Mn^2+^ concentration. The higher overall Mn^2+^ concentration in *bicat3* indicates that *bicat3* plants are more efficient in Mn^2+^ uptake from media with very low Mn^2+^ concentrations and that the increase in cellular Mn^2+^ is channeled toward the chloroplasts. This observation might suggest that BICAT3 is involved in or upstream of signaling events connected to plasma membrane Mn^2+^ uptake.

The improved photosynthesis and higher Mn^2+^ concentration might sound positive at first glance, yet, *bicat3* plants showed a reduction in cell size due to cell wall defects. These cell wall defects in *bicat3* are likely the result of reduced glycosyltransferase activity in the Golgi and suggest that other Mn^2+^-transport proteins, like ECA3, cannot (fully) replace BICAT3 function. Surprisingly, comparing *bicat3* and *eca3* plants, He et al. 2022 found that photosynthesis was improved in *eca3* in their hydroponic experimental set-up, albeit less pronounced than in *bicat3*. This contrasts with previous findings using in vitro grown seedlings that show severely reduced chlorophyll content in *eca3* (Mills et al., 2008), suggesting decreased photosynthesis. The different observations demonstrate the complexity of ion transport and the importance of investigating plants using a diverse set of growth conditions.

Several very interesting questions arise from the characterization of *bicat3* plants. One could speculate that loss of Golgi Mn^2+^ transport might lead to a signaling mechanism in the Golgi, which detects insufficient Mn^2+^ or activity alterations dependent on Mn^2+^ processes. Through yet unknown mechanisms, this signal seems to result in increased (capacity of?) cellular Mn^2+^ uptake at the plasma membrane under Mn^2+^-deficient conditions, and *bicat3* plants contain more Mn^2+^ compared to the wild-type. Communication between ion transport at endomembranes and the plasma membrane has been observed for other ions, and research in this area is a field that is rapidly developing (Horaruang et al., 2020).

One could further speculate that in *bicat3* plants, which are unable to sufficiently import the now increased cytosolic Mn^2+^ into the Golgi, Mn^2+^ is then alternatively channeled into the chloroplasts, improving photosynthesis. This is speculative, and there are other plausible interpretations of the observed phenotype of *bicat3*. Yet, the higher overall Mn^2+^ concentration in *bicat3* plants demonstrates that cellular processes to increase Mn^2+^ are present in plants and more Mn^2+^ can be allocated to chloroplasts under Mn deficiency. It is yet unknown why wild-type Arabidopsis plants do not increase Mn^2+^ uptake under Mn deficiency. If we could identify the signaling mechanism for cross-talk between Golgi Mn^2+^ uptake and plasma membrane Mn^2+^ uptake without reducing Golgi Mn^2+^ content and disrupting cell wall biosynthesis, we might be able to generate plants that grow more efficiently in soils with low Mn^2+^. This would be a substantial advantage in agriculture that could lead to reduced yield losses and fertilizer usage. Expanding our knowledge on the complexity and cross-talk of ion uptake and cellular ion compartmentation is therefore an important step in this direction.

*Conflict* *of interest statement*. There is no conflict of interest.
